# HPV16 Oncoproteins Promote Cervical Cancer Invasiveness by Upregulating Specific Matrix Metalloproteinases

**DOI:** 10.1371/journal.pone.0071611

**Published:** 2013-08-13

**Authors:** Jittranan Kaewprag, Wareerat Umnajvijit, Jarunya Ngamkham, Mathurose Ponglikitmongkol

**Affiliations:** 1 Molecular Medicine Graduate Program, Faculty of Science, Mahidol University, Bangkok, Thailand; 2 National Cancer Institute, Thailand, Bangkok, Thailand; 3 Department of Biochemistry, Faculty of Science, Mahidol University, Bangkok, Thailand; University of Patras, Greece

## Abstract

Production of matrix metalloproteinases (MMPs) for degradation of extracellular matrix is a vital step in cancer metastasis. We investigated the effects of HPV16 oncoproteins (16E6, 16E6*I and 16E7), either individually or combined, on the transcription of 7 *MMP*s implicated in cervical cancer invasiveness. The levels of 7 *MMPs* reported to be increased in cervical cancer were determined in C33A stably expressing different HPV16 oncoproteins using quantitative RT-PCR and compared with invasion ability of cell lines using *in vitro* invasion and wound healing assays. Overexpression of *MMP-2* and *MT1-MMP* was detected in HPV16E6E7 expressing cells which correlated with increased cell invasion. Combination of HPV oncoproteins always showed greater effects than its individual form. Inhibition of cell invasion using a specific MMP-2 inhibitor, OA-Hy, and anti-MT1-MMP antibody confirmed that invasion in these cells was dependent on both MMP-2 and MT1-MMP expression. Depletion of HPV16E6E7 by shRNA-mediated knock-down experiments resulted in decreased MMP-2 and MT1-MMP expression levels as well as reduced invasion ability which strongly suggested specific effects of HPV oncoproteins on both MMPs and on cell invasion. Immunohistochemistry study in invasive cervical cancers confirmed the enhanced *in vivo* expression of these two MMPs in HPV16-infected cells. In addition, possible sites required by HPV16E6E7 on the *MMP-2* and *MT1-MMP* promoters were investigated and PEA3 (at −552/−540 for *MMP-2*, −303 for *MT1-MMP*) and Sp1 (at −91 for *MMP-2,* −102 for *MT1-MMP*) binding sites were shown to be essential for mediating their transactivation activity. In conclusion, our study demonstrated that HPV16E6 and E7 oncoproteins cooperate in promoting cervical cancer invasiveness by specifically upregulating *MMP-2* and *MT1-MMP* transcription in a similar manner.

## Introduction

Persistent infection with high-risk human papillomaviruses (HPVs) is the main cause of cervical cancer, which is the second most common cancer in Thai women [1]. HPV16 is detected most often and accounts for more than 50% of cervical cancer cases worldwide [2]. Carcinogenesis due to HPV16 is attributed to the viral oncoproteins, E6 and E7, through their interactions with host cellular proteins involved in cell cycle regulation resulting in cell transformation and immortalization [3]. HPV16E7 (16E7) binds to the cell cycle protein pRb, which is subsequently subjected to degradation via a proteasome-mediated process [4], while HPV16E6 (16E6) interacts with and induces proteasome-mediated degradation of p53 [5]. Both E6 and E7 oncoproteins are also able to modulate the transcription of several host genes. Examples include the 16E6-dependent upregulation of the catalytic subunit of human telomerase reverse transcriptase (*hTERT*) and transforming growth factor β (*TGFβ* genes through specific Sp1 binding sites [6], [7] and increased expression of DEK proto-oncogene, encoding a senescence inhibitor, by E7 through a pRb family protein dependent pathway [8]. In addition to producing full-length E6 (16E6F), HPV16 also generates a truncated form of E6 (16E6*I), which promotes Dlg protein degradation [9] and transactivation of aldo-keto reductase gene [10].

The role of virus proteins on tumor invasiveness was first noted in a study demonstrating in hepatoma cell line the ability of hepatitis B virus (HBV) X protein to induce expression of matrix metalloproteinases MMP-2 and MT1-MMP (MMP-14), via a Cox 2-dependent mechanism [11]. MMPs belong to a family of zinc proteases responsible for degradation of extracellular matrix that is required for migration and metastasis of cancer cells [12]. Recent studies have indicated an association between the presence of MMPs and HPV in cervical cancer [13], [14]. Elevated expression levels of a number of MMPs (MMP-1, MMP-2, MMP-3, MMP-7, MMP-9, MMP-10, MMP-11, MMP-12, MMP-13, MT1-MMP and MMP-15) have been reported in invasive cervical carcinomas compared with normal tissues [15]–[17]. However, a correlation between HPV oncoproteins and MMP types remains controversial. Smola-Hess *et al*. demonstrated that keratinocytes that were transformed by HPV16 and HPV8 induced expression of MT1-MMP [14]. In a more recent study, the elevated HPV16E7 expression was shown to associate with an increase in pro-MMP-9 activity in organotypic culture of keratinocytes however, HPV16E6/E7 had no effect on MMP-2, -9 and MT1-MMP levels in human foreskin keratinocytes [18]. Although overexpression of several types of MMP has been observed in cervical cancer tissues, only increased levels of MMP-2 and MMP-9 were shown to correlate with poor prognosis in patients [16]. In addition, a relationship between *MMP-2* mRNA levels and cervical cancer invasiveness has been demonstrated [19]. Activation of both MMP-2 and MT1-MMP was found in squamous cervical carcinomas [16] and the generation of the active form of MMP-2 was shown to require activation by MT1-MMP [20]. This requirement is supported by the demonstration that MT1-MMP is capable of cleaving MMP-2 to form a pro-MMP-2/MT1-MMP/TIMP-2 complex, which enhances the concentration of active MMP-2 at the leading edge of invading cancer cells [21]. Although a number of MMPs have overlapping activity on a similar group of substrates, regulation of their expression levels appears to be independently regulated. *MMP-2* is constitutively expressed in many cell types, whereas cytokines regulate *MMP-9* transcription [22] and the regulation of *MT1-MMP* (constitutive or induced) remains unclear [14].

In this study, we analyzed the ability of oncoproteins from high-risk HPV16 and HPV18 to transcriptionally regulate 7 types of *MMP*s *(MMP-1, -2, -7, -9, -10, -11* and *MT1-MMP)* and to correlate MMP expression with cell invasion in cervical cancer cell lines. In addition, these results were compared with those obtained from biopsies of invasive stage cervical cancer. We speculated that high-risk HPV oncoproteins upregulated specific types of MMPs in cervical cancer cells at the transcriptional level.

## Materials and Methods

### Ethics Statement

Human tissue samples used in this study were obtained with written informed consent from the patients or their relatives. This study was approved by the Ethics Committee of National Cancer Institute, Thailand (EC 236/2011).

### Plasmid Constructs and Stably Transfected Cell Lines

HeLa, SiHa, Caski (kindly provided by P. Chambon, IGBMC, France) and C33A (ATCC: HTB-31) cervical cancer cell lines and a human embryonic kidney 293T cell line (kindly provided by P. Angeletti, University of Nebraska Lincoln, USA) were maintained at 37°C under an atmosphere of 5% CO_2_ in DMEM supplemented with 10% FBS and 1% penicillin-streptomycin cocktail (GIBCO, Invitrogen). HPV16 oncogene cDNAs, namely, 16E6 (containing both 16E6*I and 16E6F), 16E6*I, 16E7, 16E6E7 (both 16E6 and 16E7) and 16E6*IE7 (both 16E6*I and 16E7) were prepared from SiHa cells and cDNAs of HPV18E6E7 (both 18E6 and 18E7) were prepared from HeLa cells. All cDNAs were inserted into the pcDNA3 vector, and C33A cells stably expressing HPV proteins were generated and maintained as described previously [10]. Two short hairpin (sh)RNA constructs (shE6A and shE6B) containing complementary oligonucleotides designed for silencing HPV16E6 mRNA [23] and non-targeting negative control shRNA (shCtrl) were cloned into pSUPER.retro.puro (OligoEngine). Promoter constructs were generated by amplifying DNA sequences −1,721/+40, −1,001/+40 and −211/+40 of the *MMP-2* promoter and −1,277/+187, −425/+187 and −151/+187 of *MT1-MMP* promoter from C33A cells and inserted into plasmid pGL3-Basic (Promega). The following primers; −1,721/+40 *MMP-2* forward primer 5′ GAGTGCTCGAGGATCAGGCTGAAGGGCCTGG 3′, −1,001/+40 *MMP-2* forward primer 5′ GAGTGCTCGAGGAATTCGTGGAACTGAGGG 3′, −211/+40 MMP-2 forward primer 5′ GAGTGCTCGAGCGAGAGAGGCAAGTGGGG 3′, *MMP-2* reverse primer 5′ CCTGAAAGCTTCTGGATGCAGCGGAAACAA 3′, −1,277/+187 *MT1-MMP* forward primer 5′ CACCTCGAGGGAGGCTCCACAGGACCTGAAA 3′, −425/+187 *MT1-MMP* forward primer 5′ CCACTCGAGTCCCACACTCTGAGCTCCTCGTT 3′, −151/+187 *MT1-MMP* forward primer 5′ TTGCTCGAGGGCTAAAACAACCACGTCCCCA 3′ and *MT1-MMP* reverse primer 5′ CCGGGAAGCTTGGGCACTGTGGGCTCCGC 3′ were used.

### Reverse Transcription (RT)-PCR and Quantitative PCR (qPCR)

Total RNA was extracted from cells with the Illustra™ RNAspin Mini kit (GE Healthcare) and used as template for cDNA synthesis employing SuperScript®III RNaseH^-^RT Kit (Invitrogen). qPCR was performed using Stratagene Mx3000P qPCR system (Agilent Technologies) together with RBC ThermOne® Real-Time Premix (SYBR Green). Thermocycling conditions for both qPCR and RT-PCR were as follows: 10 min at 95°C; (40 cycles for RT-PCR), 30 sec at 95°C, 30 sec at 60°C and 30 sec of 72°C. Determination of hypoxanthine-guanine phosphoribosyltransferase (*HPRT*) housekeeping gene expression was included in each experiment to correct for variations in template concentrations. The amounts of PCR amplicons were analyzed using Gel Doc 2000 and Quantity One software package (Bio-Rad). The primer sets used for amplification of HPV16E6/E7, *MMP*, *TIMP-2* and internal control *HPRT* cDNAs have been previously described [10], [24], [25]. Relative expression is presented as mean± S.E.M of three independent experiments conducted in duplicate.

### Gelatin Zymography

Gelatinase activity of MMP-2 was assessed using gelatin zymography as previously described [26]. C33A stably expressing different oncoproteins were incubated in serum-free medium for 48 h before analysis. After being concentrated using an Amicon® Ultra-0.5 centrifugal filter device (Millipore), equal amounts of total protein in the spent medium from each cell line was mixed with non-reducing sample buffer (2% SDS, 10% glycerol, 62.5 mM Tris–HCl pH 6.8 and 0.01% bromophenolblue) and run on a 7.5% polyacrylamide gel containing 1 mg/ml gelatin (Sigma). After electrophoresis, gels were renatured by washing with 2.5% Triton X-100 twice for 30 min. The clear bands of hydrolyzed substrate were developed by incubation in developing buffer (50 mM Tris–HCl pH 8.0, 10 mM CaCl_2_, 1 µM ZnCl_2_, 0.02% NaN_3_ and 1% Triton X-100) at 37°C for 18 h and stained with 0.025% (w/v) Coomassie blue R250 for 1 h. The gelatinolytic activity of MMP bands was quantified by densitometric analysis (Quantity One program, Gel Doc 2000 system, Bio-Rad, USA) and presented as fold increase of the total MMP-2 (pro-MMP-2 (72 kD) and active MMP-2 (68 kD)) compared to the control pcDNA3 cells at 48 h.

### *In vitro* Invasion Assay

*In vitro* invasion assays were performed in a Transwell chamber coated on the upper chamber with 30 µg of Matrigel® (BD Bioscience). Cells (2×10^5^) in serum-free medium were seeded onto the upper chamber and DMEM containing 10% FBS was added to the lower chamber. For inhibition assays, the cells (10^4^ for SiHa and 5×10^4^ for 16E6E7) were treated with varying concentrations (0, 2, 5, 10 or 20 µM) of the MMP-2 inhibitor, *cis*-9-Octadecenoyl-N-hydroxylamide; OA-Hy (Calbiochem), or with 20 µg/ml anti-MT1-MMP antibody (ab78738, Abcam) in the upper chamber with 1% FBS for SiHa and 2% FBS for 16E6E7 in the lower chamber. After incubation for 24 h (or 12 h in the inhibition assays) for 16E6E7 and 12 h (or 7 h in the inhibition assays) for SiHa, cells in the lower chamber were fixed with 4% paraformaldehyde and stained with 0.5% crystal violet. Invasion activity was determined by counting the numbers of invaded cells in five random fields and results are presented as mean ± S.E.M of stained cells relative to the controls, vehicle or the non-targeting shRNA plasmid or untreated cells.

### Wound Healing Assay

Cells were grown until confluence and an artificial wound was generated using P-200 pipette tip. DMEM containing 100 µg of Matrigel® (BD Bioscience) was added to cells and allowed to polymerize for 15 min. The numbers of cells migrating into the scratched region were counted at 0 and 48 h after incubation and reported as percent cells in the scratched region compared to control cells.

### Luciferase Activity Assay

C33A cells expressing HPV16E6E7 were transiently transfected with pGL3-Basic luciferase vector constructs containing *MMP-2* and *MT1-MMP* promoters as described above. Transfected cells were lysed and assayed for the luciferase activity as reported previously [10].

### Western Blot Analysis

C33A cells expressing HPV16 oncoproteins were lysed in Lysis-M Reagent, EDTA-free, with complete Protease Inhibitor Cocktail (Roche). The proteins were separated on 10% SDS-PAGE and transferred to PVDF membranes. After blocking with 10% non-fat dry milk in PBST, the membranes were incubated with mouse anti-p53 (sc-126, Santa Cruz Biotechnology at 1∶5,000), mouse anti-MT1-MMP (ab78738, Abcam at 1∶1,500), mouse anti-TIMP-2 (ab1828, Abcam at 1∶1,000), or mouse anti-GAPDH (sc-166574, Santa Cruz Biotechnology at 1∶2,000) antibodies at 4°C overnight followed by incubation with a secondary HRP-conjugated sheep anti-mouse IgG (GE healthcare) (1∶3,000) for 2 h at room temperature. The results were visualized using the Pierce ECL system (Thermo Scientific), with GAPDH as a loading control. Protein levels were quantitated using densitometry (Quantity One program, Gel Doc 2000 system, Bio-Rad) and results are presented as the fold increase of protein (both pro- and mature forms) compared with vehicle control after normalization against GAPDH.

### Fluorescent Immunohistochemistry

All clinical investigation has been conducted according to the declaration of Helsinki and Good Clinical Practice. Paraffin-embedded tissue sections from 26 invasive cervical carcinomas, three cervical intraepithelial neoplasia (one each of CIN 1, CIN 2, and CIN 3), and one adenomyosis were obtained from the National Cancer Institute (NCI), Thailand. All tissues were analyzed by NCI pathologists and made anonymous before being submitted to the study. Antigens were retrieved by microwaving the samples in Tris-EDTA buffer (10 mM Tris base pH 9.0, 1 mM EDTA, 0.05% Triton X-100) for 5 min and endogenous peroxidase activity was inhibited by treatment with 3% H_2_O_2_ for 10 min. Fluorescent immunohistochemistry was performed by incubating 4°C overnight with primary mouse anti-MMP-2 and anti-MT1-MMP monoclonal antibodies (42-5D11 and 114-6G6 respectively; Chemicon) at a 1∶100 dilution followed by incubating with secondary Alexa Fluro 647-labelled goat anti-mouse IgG antibody (1∶400 dilution; Invitrogen). Hoechst 33258 (1∶1,000 dilution; Invitrogen) was used for nuclear staining. Sections were mounted with VECTASHIELD® anti-fade medium (Vector Laboratories) and fluorescent signals were measured using an Olympus FV1000 confocal microscope equipped with ImageJ software. MMP-2 and MT1-MMP expression were scored as positive or negative after subtracting with baseline controls without primary antibody treatment giving the number of stained cells of ≥1% or 0% respectively.

## Results

### Effects of HPV and HPV Oncoproteins on *MMP* Expression and Invasion Ability

**Figure 1:**
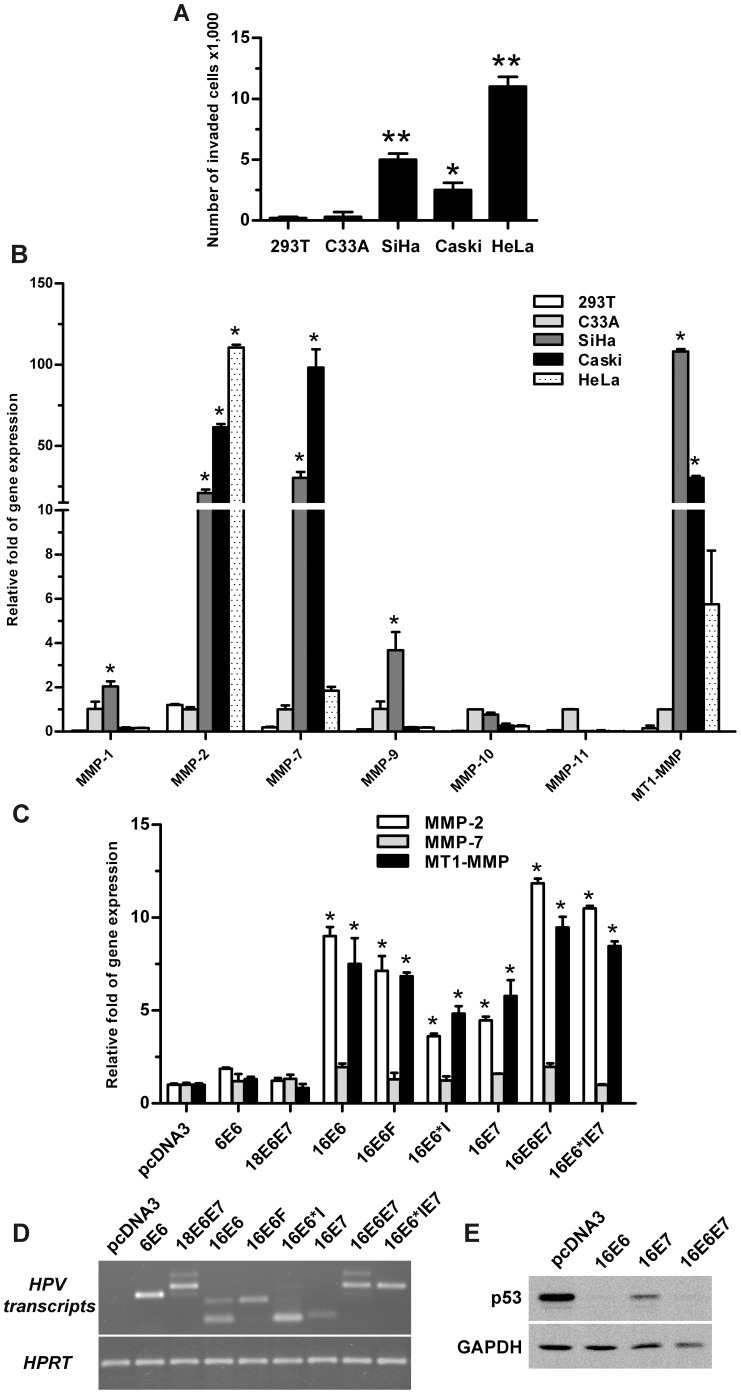
*In vitro* invasion and *MMP* expression of HPV positive, HPV negative and HPV oncoprotein expressing C33A. (A) Quantitation of invasion using migration through filters coated with Matrigel for HPV positive (SiHa, Caski and HeLa) and negative (C33A and 293T) cells presenting as numbers of invading cells. (B) Relative expression of *MMP-1*, *-2*, *-7*, *-9*, *-10*, *-11* and *MT1-MMP* measured in cell lines using qPCR. Data was normalized to C33A. *:*p*-value<0.05 relative to C33A. (C) Relative expression of *MMP-2, -7* and *MT1-MMP* in C33A cells stably expressing different HPV16 (16E6, 16E6F, 16E6*I, 16E7, 16E6E7, 16E6*IE7), HPV18 *(18E6E7)* and low-risk HPV6 *(6E6)* oncoproteins as measured by qPCR and normalized to pcDNA3. *p-value<0.05 compared to pcDNA3 expressing cells. (D) The level of HPV transcripts compared to the control, HPRT, in each cell line as shown by RT-PCR. (E) P53 degradation activity of 16E6, 16E7 and 16E6E7 was compared by western blot analysis. GAPDH was used as loading control.

Invasion ability of cervical cancer cell lines harboring HPV genome (SiHa with HPV16, Caski with HPV16 and HeLa with HPV18) were compared with HPV-negative C33A and 293T cells. All HPV-positive cell lines clearly displayed an ability to invade, whereas invasiveness of HPV-negative cell lines was minimal (Fig. 1A). Higher invasion ability was observed with SiHa (5,000 cells) and HeLa (11,000 cells) compared to Caski cells (2,500 cells) (Fig. 1A). The expression levels measured using qPCR of the 7 *MMP*s (*MMP-1, -2, -7, -9, -10, -11* and *MT1-MMP)* in these five cell lines showed a correlation between the presence of HPV and an increase in *MMP* transcripts only of *MMP-2*, *-7* and *MT1-MMP* (Fig. 1B). Upregulation of *MMP-2* expression was highly detected in both HPV16- and HPV18-positive cells compared to HPV-negative cells, with the highest activity in HPV18-positive HeLa cells. *MMP-7* and *MT1-MMP* transcript levels were significantly increased in HPV16-positive SiHa and Caski cells, but only slightly elevated in HeLa cells (Fig. 1B). However, there were no elevation of *MMP-10* and *-11* transcripts in any HPV-positive cells (Fig. 1B). These results indicate that expression of *MMP-2*, *-7* and *MT1-MMP* are more likely to occur in cells harboring HPV, particularly HPV16.

In order to examine the relationship between high expression of *MMP-2, -7* and *MT1-MMP* and HPV oncoproteins, qPCR was employed to monitor *MMP* transcript levels in C33A cells stably expressing 16E6, 16E6F, 16E6*I, 16E7, 16E6E7, 16E6*IE7, 18E6E7 and (low-risk) HPV6E6. 16E6F, 16E6*I and 16E7 were able to induce an increase in *MMP-2*, *MT1-MMP* but not *MMP-7* transcripts, compared to pcDNA3-transfected control cells (Fig. 1C). C33A cells expressing 16E6*I showed the lowest *MMP-2* and *MT1-MMP* transcripts among the HPV16 oncoproteins tested. In cells expressing 16E6F and 16E6*I (i.e. 16E6-expressing cells) or 16E6*I and 16E7 (i.e. 16E6*IE7-expressing cells) or 16E6F, 16E6*I and 16E7 (i.e. 16E6E7-expressing cells) there were significant increases in *MMP*s transcripts, particularly those of *MMP-2* and *MT1-MMP* (Fig. 1C). However, in C33A cells expressing 18E6E7, transcript levels of *MMP-2*, *MMP-7* and *MT1-MMP* were not different from those in pcDNA3 expressing cells, and as expected, there were no changes of *MMP* transcripts in low-risk HPV6E6 expressing cells compared to pcDNA3 expressing cells. These results clearly demonstrate that HPV16 but not HPV18 oncoproteins specifically enhance the expression of *MMP-2* and *MT1-MMP*, but not of *MMP-7*. The levels of HPV16 oncogene transcripts in C33A cells as shown by RT-PCR in Fig. 1D indicated that *MMP* inducing activity correlated more with types but not dosage of HPV oncoproteins. The functional activities of HPV oncoproteins in degrading p53 proteins were also demonstrated in 16E6, 16E7 and 16E6E7 expressing cells (Fig. 1E).

### Invasiveness, Gelatinase Activity and MMP Expression in C33A Cells Expressing HPV16 Oncoproteins

**Figure 2:**
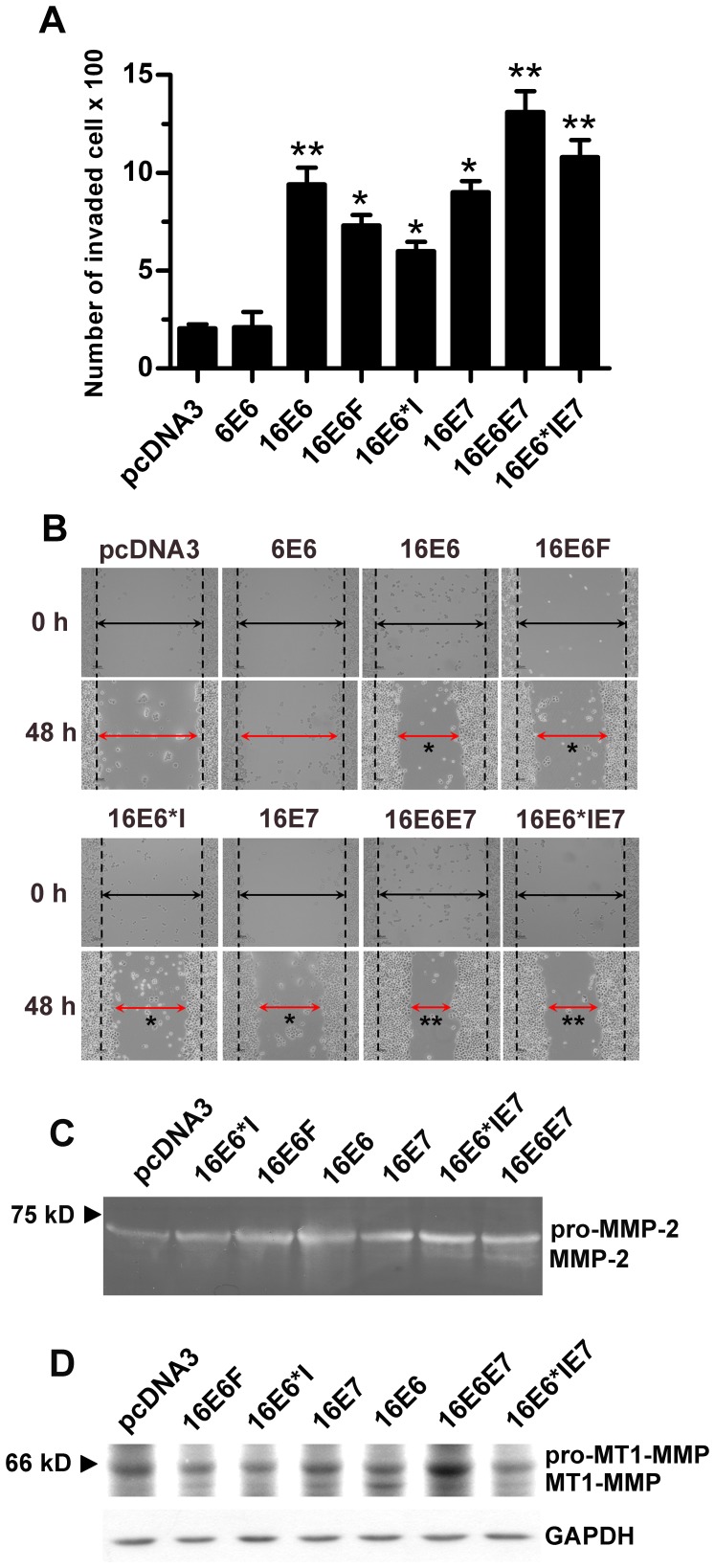
Invasion ability, MMP expression and activity of C33A cells stably expressing different HPV16 oncoproteins. (A) *In vitro* invasion and (B) wound healing assays for C33A cells expressing different HPV16 oncoproteins. (C) A representative gelatin zymographic gel showing MMP-2 activity normalized with equal amounts of loading protein (30 µg). Bands corresponding to the gelatinolytic activity of MMP-2 were quantified by densitometric analysis and compared with those obtained from control pcDNA3 expressing cells. (D) Western blot analysis of MT1-MMP using anti-MT1-MMP antibody (ab78738) at 1∶1,500 dilution with GAPDH as loading control. *p-value<0.05, **p-value<0.01.

We further determined whether increases in *MMP-2* and *MT1-MMP* transcripts translate into enhanced invasiveness of HPV16-oncogene expressing cells utilizing both *in vitro* invasion (Fig. 2A) and wound healing assays (Fig. 2B). HPV16 oncoprotein expressing C33A were more invasive in both assays than control HPV6E6 expressing and pcDNA3-transfected C33A, with cells expressing a combination of E6F, E6*I and E7 (16E6E7) having the highest invasion capability (Fig. 2A and B). As these cells have increased *MMP-2* and *MT1-MMP* transcripts, they were subjected to gelatin zymography using equal amount of proteins (30 µg) to determine if elevated MMP activity was present. Medium from all C33A cells showed the presence of a gelatinase, corresponding to pro-MMP-2 (∼72 kD) and MMP-2 (∼62 kD) [14], with more intense MMP-2 bands present in cells expressing 16E6, 16E6*IE7 and 16E6E7 (Fig. 2C). Cells containing low-risk HPV6E6 showed similar gelatinase activity to the control pcDNA3 containing cells (data not shown). The gelatin lysis band in gelatin zymographies has been ascertained by adding 5 mM of disodium EDTA, a gelatinase inhibitor, into the reaction and running onto a separate gel. Disappearance of the expected zymographic bands confirmed gelatinase activity of those lysis bands (data not shown). It is worth noting that the combined presence of 16E6*I and 16E6F in 16E6 enhances active MMP-2 levels compared with cells expressing 16E6F or 16E6*I alone (Fig. 2A, 2B, 2C), suggesting an additive effect between the two forms of E6 in stimulating *MMP-2* expression and thereby promoting cell invasion. The protein levels of MT1-MMP in these cells were also determined by western blot analysis using anti-human MT1-MMP antibody with GAPDH as a control. The fold increase of MT1-MMP expression was calculated from total MT1-MMP (pro-MT1-MMP and MT1-MMP) with the vector control set as 1. As shown in Fig. 2D, all cells containing HPV16 proteins expressed both pro- (∼66 kD) and mature MT1-MMP (∼57 kD) with the greatest band intensity of pro-MT1-MMP in 16E6E7 (2.5×, total MT1-MMP) and of mature MT1-MMP in 16E6 (2.3×, total MT1-MMP) whereas the vector control showed only the pro-MT1-MMP. These observations revealed that the invasiveness of HPV16 oncoprotein expressing cells correlated with MMP-2 activity and MT1-MMP expression.

### Effect on Invasiveness of HPV16E6 Knock-down Cells

**Figure 3:**
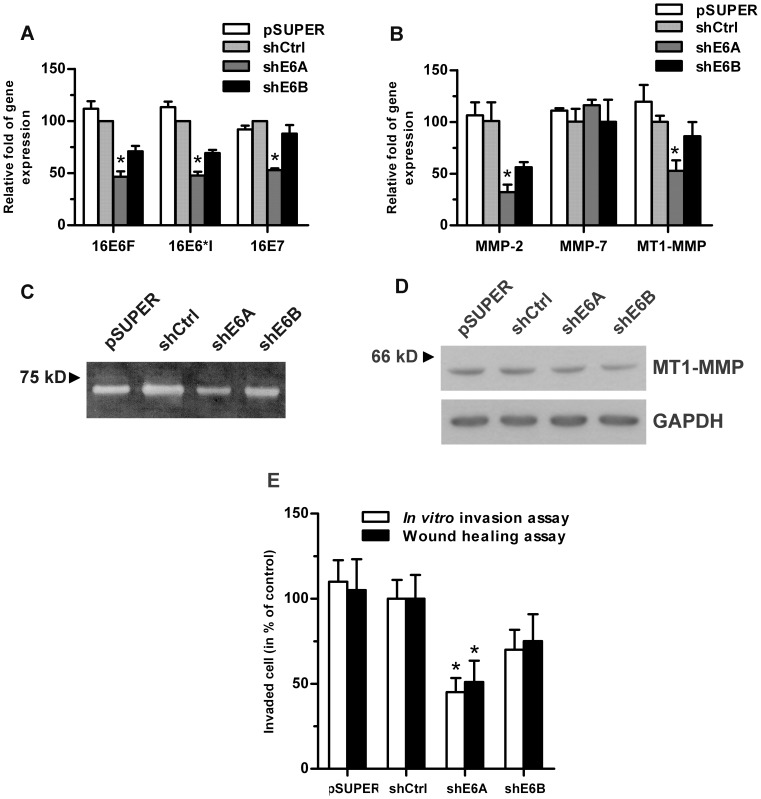
HPV oncogenes, *MMP* transcripts and MMP protein levels in SiHa cells transfected with shRNA directed at E6 oncogene. (A) RT-PCR of 16E6F, 16E6*I, and *16E7* transcripts normalized to the control, *HPRT*. SiHa cells transfected with non-targeting shCtrl and vehicle control pSUPER were used as negative controls. shE6A and shE6B are two different shRNAs used in knock-down experiments. (B) qPCR of *MMP-2*, *-7* and *MT1-MMP* transcripts and (C) MMP-2 activity as assayed by gelatin zymography, with equal amounts of protein (20 µg), in shRNA transfected cells. (D) MT1-MMP protein in knocked down cells as determine by western blot analysis with GAPDH as loading control. (E) Invasion ability of SiHa cells transfected with different shRNAs as determined by *in vitro* invasion and wound healing assays. Relative fold of cell invasion with untreated cells were set as 1 is reported. *p-value<0.05 compared to shCtrl cells.

To confirm that HPV16 oncoproteins cause upregulation of *MMP-2* and *MT1-MMP* expression, E6 oncogenes in HPV16-positive SiHa cells were knocked down using two shRNAs (shE6A and shE6B) targeting different sites of the 16E6 mRNA [23]. Using RT-PCR, levels of HPV16E6F, 16E6*I and 16E7 transcripts in SiHa cells were shown to be reduced by ∼50% after transfection with shE6A, whereas only a 10–30% reduction was detected in cells transfected with shE6B (Fig. 3A). Only *MMP-2* and *MT1-MMP* but not *MMP-7* transcripts were reduced in E6 knock-down SiHa cells (Fig. 3B). When equal amounts of protein (20 µg) from the conditioned medium was assayed by gelatin zymography, it was observed that MMP-2 activity in shE6A-transfected cells was decreased compared to pSUPER and shCtrl transfected control cells (Fig. 3C). Cell lysates (30 µg protein each) analyzed using western blots to assess MT1-MMP levels with GAPDH as control, revealed slight decreases (∼30% and ∼40% reduction in shE6A and shE6B respectively) of MT1-MMP levels in both knock-down cells compared to shCtrl controls (Fig. 3D). Interestingly, MT1-MMP proteins detected in SiHa cells were mainly the mature MT1-MMP (57 kD). Consistent with these findings, both shRNA-transfected cells, particularly shE6A, had significantly reduced invasiveness (∼48%) and wound healing ability (∼50%), compared to shCtrl cells (Fig. 3E). Thus, ablation of HPV16E6/E7 oncoproteins reduces MMP-2 activity, MT1-MMP expression and finally invasiveness of HPV16-positive SiHa cells.

### Reduced Invasiveness by Specific Inhibitors to MMP-2 and MT1-MMP

**Figure 4:**
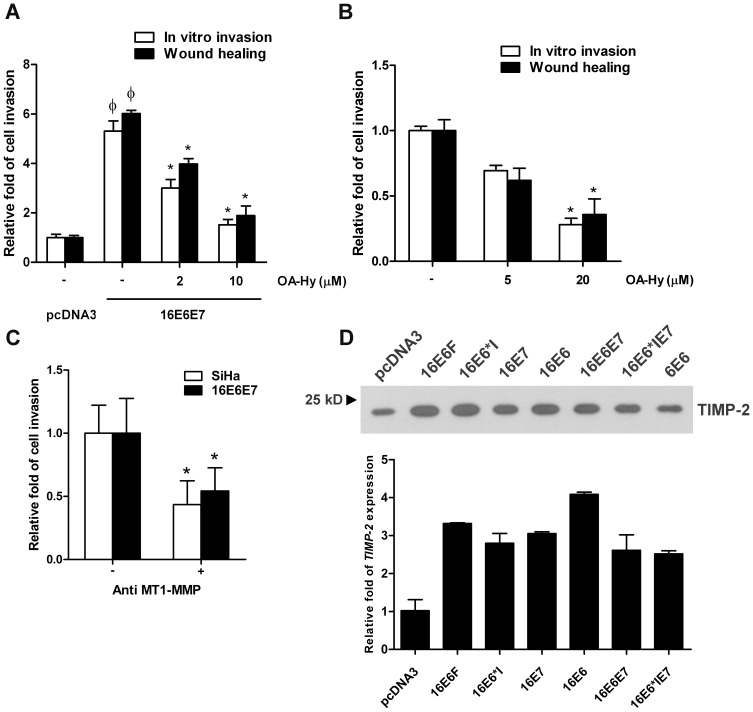
Inhibition of cell invasion by specific inhibitors for MMP-2 and MT1-MMP and assay of TIMP-2. (A) Cell invasion as determined using *in vitro* invasion and wound healing of HPV16E6E7 expressing cells and (B) SiHa cells treated with various concentrations of MMP-2 inhibitor, OA-Hy. (C) The same cell lines were also treated with anti-MT1-MMP antibody (20 µg/ml). Relative fold of cell invasion with pcDNA3 vehicle control or untreated cells set as 1 at φ*p*<0.05 compare to pcDNA3 and *p-value<0.05 compare to untreated cells is shown. (D) TIMP-2 protein (normalized with equal loading protein of 5 µg) was detected using western blot analysis with an anti-TIMP-2 antibody (abcam, 1∶1,000 dilution) and TIMP-2 transcripts were measured by qPCR (normalized to HPRT) for HPV16 oncoprotein expressing cells.

To determine whether selective inhibition of endogenous MMP-2 or MT1-MMP impair invasion ability, both SiHa and 16E6E7 expressing C33A cell lines were treated with OA-Hy, a specific inhibitor for MMP-2, (Fig. 4A, 4B) and anti-MT1-MMP, a specific antibody for the catalytic domain of MT1-MMP (Fig. 4C). Our results clearly showed that treatment of OA-Hy resulted in a significant reduction in cell invasion for both 16E6E7 (∼70% at 10 µM) (Fig. 4A) and SiHa cells (∼65% at 20 µM) (Fig. 4B) in a dose-dependent manner. Similarly, MT1-MMP inhibition using the specific antibody clearly reduced invasiveness (∼50%) of both cell lines compared to untreated cells (Fig. 4C). Our experiments confirmed that invasion of HPV16-infected cells was dependent on MMP-2 and MT1-MMP expression. Since it has been known that the formation of a MT1-MMP/TIMP-2/pro-MMP-2 complex is necessary for the activation of pro-MMP-2 by MT1-MMP on the cell surface, the levels of TIMP-2 expression in all HPV16 oncoprotein expressing cells were also investigated. Interestingly, TIMP-2 expression was found to be slightly increased both at the mRNA (∼3×) and protein (∼2×) levels in all cells as compared to the control pcDNA3 with the exception of the low-risk HPV6E6 that exhibited TIMP-2 expression comparable to the empty vector (Fig. 4D). Whether HPV oncoproteins directly enhanced the expression of TIMP-2 remains to be determined.

### Transcriptional Upregulating Activity of HPV16 Oncogenes on *MMP-2* and *MT1-MMP* Promoter

**Figure 5:**
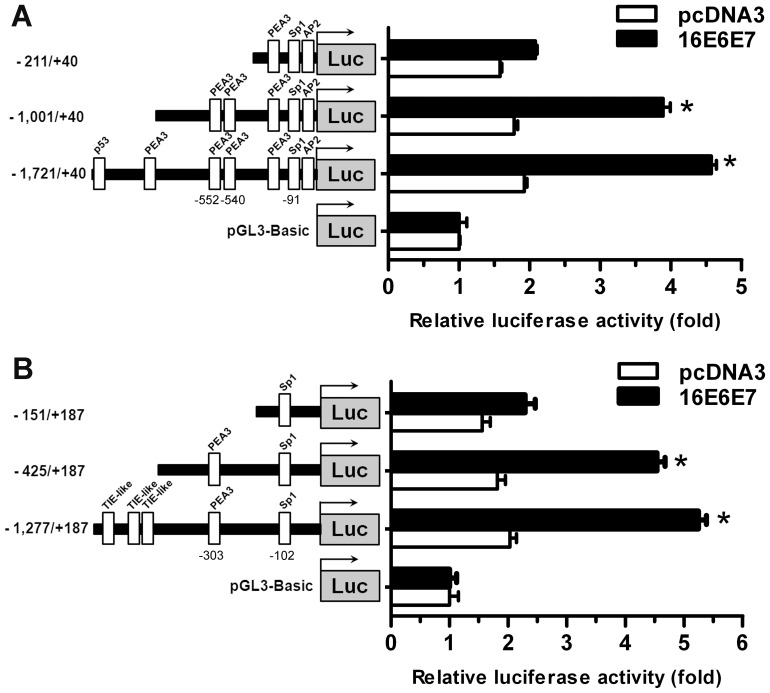
Transcriptional activation activity of HPV16E6E7 on MMP promoters. (A) *MMP-2* and (B) *MT1-MMP* promoter constructs in C33A cells expressing HPV16E6E7. The luciferase activities of three constructs of each promoter were compared with that of the pGL3-Basic vector (set as 1). *p-value<0.05 compared to pcDNA3.

Promoter constructs containing 5′-upstream sequences, namely, −1,721/+40, −1,001/+40 and −211/+40 from *MMP-2* promoter and −1,277/+187, −425/+187 and −151/+187 from *MT1-MMP* promoter fused with luciferase reporter gene in plasmid pGL3-Basic were generated. Each construct was subsequently transfected into C33A stably expressing HPV16E6E7. In the presence of HPV16E6E7, the luciferase activity of the −1,721/+40 *MMP-2* construct was shown to be comparable to that of −1,001/+40 displaying 4.6 and 3.9 fold greater expression than that of the control cells transfected with the empty pGL3-Basic vector or ∼2-fold increase of both promoter activities compared to the basal level in pcDNA3 expressing cells (Fig. 5A). Similar results were obtained from *MT1-MMP* promoter constructs (Fig. 5B). These results suggest that sequences responsible for mediating HPV16 oncoprotein activity should reside within −1,001/+40 and −425/+187 regions of *MMP-2* and *MT1-MMP* promoters, respectively. Both contained PEA-3 (at −552 and −540 for *MMP-2* and −303 for *MT1-MMP*) and Sp1 (at −91 for MMP-2 and −102 for *MT1-MMP*) binding sites suggesting that HPV16 oncoproteins exert their transactivation activity through these sites in a similar manner. The lack of p53 and the remote PEA-3 binding sites in *MMP-2* promoter and TIE-like sequences in *MT1-MMP* promoter constructs indicated that these sites were not responsible for mediating the effect of HPV oncoproteins. However, the smallest constructs of *MMP-2* promoter (-211/+40) containing one each of the Sp1, AP2 and PEA-3 binding sites and of *MT1-MMP* promoter (-151/+187) containing only Sp1 did not exhibit clear luciferase activity indicating that these binding sites were not sufficient to upregulate *MMP-2* and *MT1-MMP* expression in response to the virus proteins.

### Expression Levels of MMP-2 and MT1-MMP in HPV16-infected Invasive Cervical Cancer Biopsies

**Figure 6:**
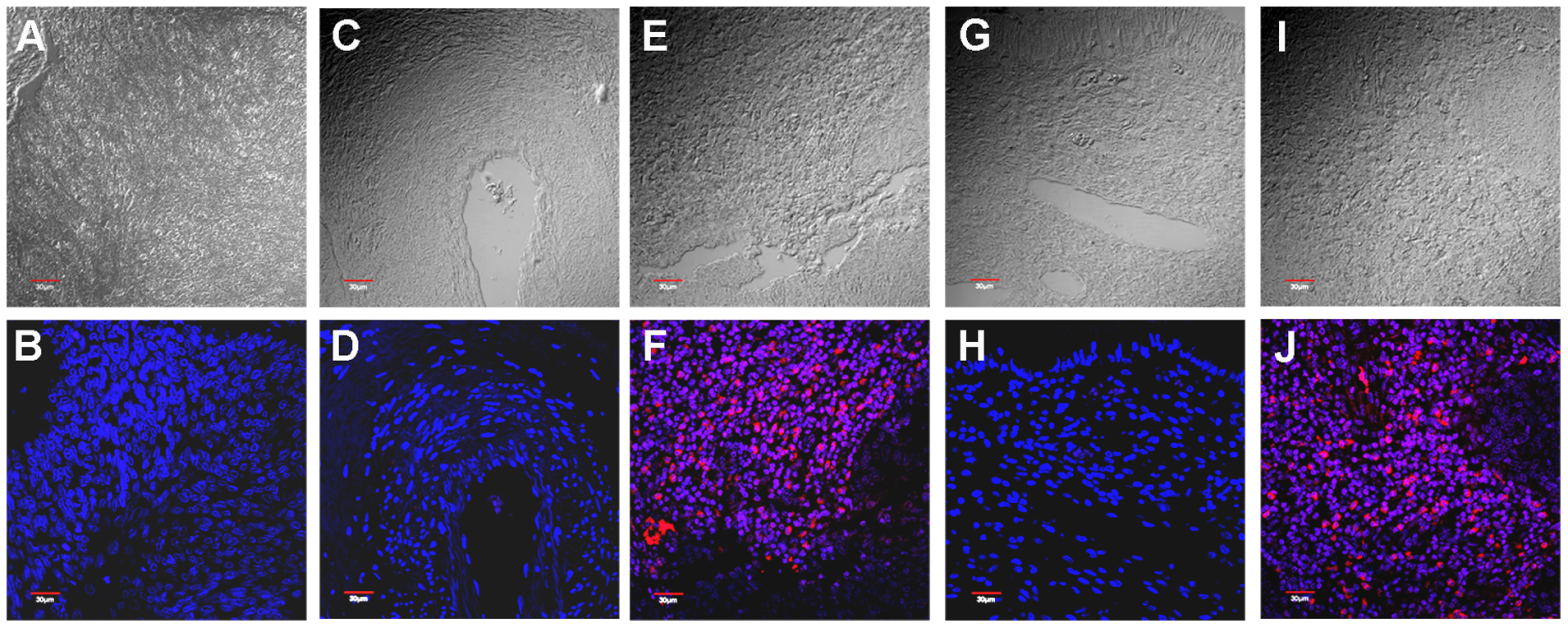
Representative phase contrast and fluorescent images of MMP-2 and MT1-MMP expression in cervical cancer tissues. Presence of MMP-2 and MT1-MMP are shown in red (Alexa Fluor 647) and cell nuclei in blue (Hoechst 33258). (A, B) no primary antibodies; (C, D) and (G, H) negative staining of MMP-2 and MT1-MMP respectively; (E, F) and (I, J) positive staining of MMP-2 and MT1-MMP respectively.

Based on the above results, we surmised that invasive cervical cancers in patients with HPV16 infection would have elevated levels of MMP-2 and MT1-MMP. A total of 30 samples of paraffin-embedded sections from cervical tissues, 15 of which were positive for HPV16 and 26 diagnosed as invasive cervical carcinomas, were examined for MMP-2 and MT1-MMP expression using fluorescent immunohistochemistry (Fig. 6). HPV genotyping was determined by PCR-Elisa [27] and confirmed by sequencing of E6 DNA (unpublished data). MMP-2 and MT1-MMP were detected in all HPV16-positive samples except for one sample that had been diagnosed as non-invasive carcinoma *in situ* CIN 2 (Table 1). Positive MMP-2 and MT1-MMP expression was therefore shown in 92% and 100% respectively of 14 invasive cervical carcinomas with positive HPV16 infection. Both MMP-2 and MT1-MMP were detected in the same samples except for one in which only MT1-MMP was detected (Table 1). There is a significant correlation between HPV16 infection and MMP-2 (87%) and MT1-MMP (93%) expression regardless of pathological staging, with *p*-values of 0.0009 and 0.0002 respectively (Table 2), which was not evident in tissues containing other HPV types or without HPV since the majority of these (73% for both) did not display MMP-2 nor MT1-MMP expression. These findings demonstrate that invasiveness of HPV16-positive cervical cancer is associated with increased expression levels of both MMP-2 and MT1-MMP, which could be due to HPV16 oncoproteins.

**Table 1:** Fluorescent immunohistochemistry of MMP-2 and MT1-MMP in invasive cervical cancers and their HPV typing.

Sample No.	HPV typing	Pathological diagnosis	MMP-2	MT1-MMP
1	16	CIN 3	+	+
2	16	CIN 2	–	–
3	16, 33, 58	Invasive	+	+
4	16	Invasive	+	+
5	16, 52	Invasive	+	+
6	16	Invasive	+	+
7	16	Invasive	+	+
8	16, 66	Invasive	+	+
9	16, 18, 45, 58, 70	Invasive	–	+
10	16	Invasive	+	+
11	16	Invasive	+	+
12	16	Invasive	+	+
13	16	Invasive	+	+
14	16	Invasive	+	+
15	16	Invasive	+	+
16	–	Adenomyosis	–	–
17	33, 35, 58	CIN 1	–	–
18	18	Invasive	+	+
19	33	Invasive	–	–
20	26	Invasive	–	–
21	58	Invasive	–	–
22	33, 58	Invasive	+	+
23	58, 70	Invasive	–	–
24	56	Invasive	–	–
25	33, 35, 45	Invasive	–	–
26	–	Invasive	+	+
27	35, 52, 66	Invasive	–	–
28	–	Invasive	–	–
29	58	Invasive	+	+
30	18	Invasive	–	–

CIN 1, 2, 3 are various stages of control carcinoma *in situ*. Adenomyosis represents normal uterine control.

**Table 2:** Correlation between HPV16, MMP-2 and MT1-MMP results in cervical cancer tissues.

		HPV16 positive (n = 15, 50%)	HPV negative or other HPV positive (n = 15, 50%)	*P*-value
**MMP-2**	Positive	13 (87%)	4 (27%)	0.0009*
	Negative	2 (13%)	11 (73%)	
**MT1-MMP**	Positive	14 (93%)	4 (27%)	0.0002*
	Negative	1 (7%)	11 (73%)	

* Statistically significant by chi-square test.

## Discussion

Overexpression of various MMPs in particular MMP-2 (gelatinase A), -9 (gelatinase B) and MT1-MMP (collagenase), has been detected in a majority of squamous cervical carcinomas, and these enzymes have been implicated in cancer progression and poor prognosis [15]–[17]. MMP-2 and MT1-MMP facilitate tumor invasion, as both are able to degrade extracellular matrix (ECM) as well as non-ECM substrates [28]. In addition, MT1-MMP increases vascular epidermal growth factor expression and sheddase activity of Mucin-1, thereby promoting tumor progression [29], [30]. Induction of MMP-2 and MT1-MMP expression by HBV X protein has been proposed to be mediated by a Cox-2 dependent mechanism [11], but a Cox-2 inhibitor (PTPBS) has no effect on MMP-2 and MT1-MMP levels in HPVE7-expressing HaCaT cells [14]. Thus it is unclear how a virus induces MMP-2 and MT1-MMP expression.

Although HPV16E7 increases MT1-MMP activity in 16E7-transfected keratinocytes by raising *MT1-MMP* transcription [14], the role of 16E6 in this regards has not until now been investigated. Moreover, the effect of HPV oncoproteins on *MMP-2* at the transcriptional level has never been reported. This study demonstrates that in addition to the known roles of HPV16 oncoproteins [31], these oncoproteins promote invasiveness of cervical cancer cells through transcriptional upregulation of both *MMP-2* and *MT1-MMP* and a combination of HPV16 oncoproteins significantly stimulates *MMP-2* and *MT1-MMP* expression and cell invasion more than individual 16E6F, 16E6*I or 16E7. Furthermore, knock-down experiments using transfected shRNAs directed against HPV16 oncogene (16E6F, 16E6*I and 16E7) transcripts in HPV16-positive SiHa cells resulted in a reduction of both *MMP-2* and *MT1-MMP* transcripts, MT1-MMP protein level, MMP-2 activity as well as *in vitro* invasiveness and wound healing ability. The dependence of cell invasion on MMP-2 and MT1-MMP was ascertained when invasion ability of both SiHa and 16E6E7 cells were impaired after treatment either with specific MMP-2 inhibitor or with MT1-MMP antibody. More importantly, an increase in immunochemical-reactive MMP-2 and MT1-MMP was detected in 92% and 100% respectively, of invasive cervical cancer biopsies positive for HPV16 highlighting the involvement of HPV16 in cervical cancer progression.

In addition, it was found that TIMP-2 levels (both mRNA and protein) were moderately increased in all HPV16 oncoprotein transfected cells when compared to the vector control. This observation prompted us to surmise that the increase of TIMP-2 levels in these cells might increase the chance of activating MMP-2 by MT1-MMP and provide a proper ratio between enzymes and its inhibitor facilitating the formation of ternary complex requisite for MMP-2 activation and cell invasion. Previous studies have also shown that the expression of MT1-MMP, MMP-2 and TIMP-2 correlated well in most, if not all, cervical cancer cell lines [32]. In particular, the HPV16 containing cell line, SiHa, exhibited remarkable expression of all three of these genes. Our observation was also supported by the findings of Asha Nair *et al*. which reported that TIMP-2 levels were increased during tumor progression in the uterine cervix [33]. However our results are in contrast with Branca *et al.* that demonstrated down regulation of TIMP-2 in invasive cervical carcinomas [34]. These contradictory data suggested that dual functions (activator and inhibitor) of TIMP-2 might be differentially regulated at the level of expression by different factors. It is of interest to address whether infection by other types of HPV in cervical tissues contributes to the difference of TIMP-2 expression. The possibility that MMP-2 activity in these HPV protein containing cell lines was affected by other MMP inhibitors such as RECK, testican or the Cupin family proteins [18], [35], [36] cannot be excluded.

The similar effect of each type of HPV16 oncoproteins and their combinations on *MMP-2* and *MT1-MMP* gene expression suggests that virus oncoproteins utilize a common controlling mechanism. Both *MMP-2* and *MT1-MMP* promoters have no TATA box but contain binding elements for Sp1 family and PEA3, a member of Ets family, located within a 500 bp upstream flanking region of both promoters [37]–[39]. The lack of a TATA box in these two *MMP* promoters emphasizes the interplay among different combinations of transcription factors in the regulation of their expression. The Sp1 binding site is essential for basal transcriptional activity of both *MMP*s as only ∼10% and ∼30% of transcription activity is observed when Sp1 at −102 on *MT1-MMP* in human fibrosarcoma HT-1080 and at −91 on *MMP-2* in astroglioma cells is mutated respectively [38], [39]. The role of PEA3 in regulating *MMP-2* and *MT1-MMP* expression has been demonstrated previously and inhibition of PEA3 expression using siRNA reduced MT1-MMP level by 50% in ovarian cancer cells [37], [40]. Binding of PEA3 to the *MT1-MMP* promoter in ovarian tumor cells is mediated through epidermal growth factor receptor (EGFR) signaling pathway [37]. However, activation of EGFR signaling pathway downregulated MMP-2 synthesis in SiHa cells [41]. Thus, it seems unlikely that HPV16 oncoproteins are acting through the EGFR signaling pathway in cervical cancer cells.

Using the *MMP-2* promoter deletion constructs, we have demonstrated that both Sp1 (at −91) and double PEA3 (at −552 and −540) binding sites on *MMP-2* promoter are necessary for upregulating activity of HPV16E6E7. Similarly, the study using the *MT1-MMP* promoter constructs showed the significance of Sp1 at −102 and PEA-3 binding sites at −303. Interaction between PEA3 and Sp1 has been reported in embryonal carcinoma P19 cells and shown to be necessary for upregulating the *T* gene required for induction of embryonic differentiation [42]. The findings that the Sp1 binding site is required for 16E6 to stimulate *TGF-β1*, *VEGF* and *hTERT* expression [6], [7], [43] and that E1A (similar in structure and function to E7, [44]) regulates the activity of E1AF (PEA3) [45] supports the linkage of these transcription factors to HPV oncoprotein activities. Therefore, it is conceivable to conclude that HPV16 oncoproteins act together on both *MMP-2* and *MT1-MMP* promoters and their transcriptional activating activity is mediated through the PEA3 and Sp1 binding sites. However, it is presently unknown if 16E6 or 16E7 could directly bind to PEA-3.

The high ability to invade cervical tissues and ultimately become invasive cervical carcinomas facilitated by MMPs is likely to have a clinical impact for treatment of the disease. The large numbers of MMPs have raised questions regarding redundancy in their function and control. Understanding of the different kinds of control mechanisms operating for each type of MMPs would help in designing appropriate therapeutic interventions. In this respect, the current study is the first to demonstrate that E6 and E7 oncoproteins from HPV16, but not HPV18, act in concert to control transcription of *MMP-2* and *MT1-MMP*, most likely through Sp1 and PEA3 binding sites and involve in cell invasion ability. Thus, infection with high-risk HPV16 not only leads to carcinogenesis of the cervix, but also contributes via 16E6 and 16E7 oncoproteins to development of invasiveness and poor prognosis of cervical cancer.
